# Expectations and perceived barriers to widespread implementation of e‑Health in cardiology practice: Results from a national survey in the Netherlands

**DOI:** 10.1007/s12471-018-1199-9

**Published:** 2018-11-28

**Authors:** R. W. Treskes, T. X. Wildbergh, M. J. Schalij, R. W. C. Scherptong

**Affiliations:** 1 Department of Cardiology, Leiden University Medical Center, Leiden, The Netherlands; 2 Department of Cardiology, Meander Medical Center, Amersfoort, The Netherlands

**Keywords:** e-Health, general cardiology, implementation

## Abstract

**Introduction:**

Expectations of physicians concerning e‑Health and perceived barriers to implementation in clinical practice are scarcely reported in the literature. The purpose of this study was to assess these aspects of cardiovascular e‑Health.

**Methods:**

A survey was sent to members of the Netherlands Society of Cardiology. In total, the questionnaire contained 30 questions about five topics: personal use of smartphones, digital communication between respondents and patients, current e‑Health implementation in clinical practice, expectations about e‑Health and perceived barriers for e‑Health implementation. Age, personal use of smartphones and professional environment were noted as baseline characteristics.

**Results:**

In total, 255 respondents filled out the questionnaire (response rate 25%); 89.4% of respondents indicated that they considered e‑Health to be clinically beneficial, improving patient satisfaction (90.2%), but also that it will increase the workload (83.9%). Age was a negative predictor and personal use of smartphones was a positive predictor of having high expectations. Lack of reimbursement was identified by 66.7% of respondents as a barrier to e‑Health implementation, as well as a lack of reliable devices (52.9%) and a lack of data integration with electronic medical records (EMRs) (69.4%).

**Conclusion:**

Cardiologists are in general positive about the possibilities of e‑Health implementation in routine clinical care; however, they identify deficient data integration into the EMR, reimbursement issues and lack of reliable devices as major barriers. Age and personal use of smartphones are predictors of expectations of e‑Health, but the professional working environment is not.

**Electronic supplementary material:**

The online version of this article (10.1007/s12471-018-1199-9) contains supplementary material, which is available to authorized users.

## What’s new?

- This is the first study to assess expectations of cardiologists of e‑Health implementation.
- Cardiologists are in general positive about the possibilities of e‑Health implementation in routine clinical care.
- Age and personal use of smartphones are predictors of expectations of e‑Health, but the professional working environment is not.

## Introduction

E-Health is a broad concept, roughly defined as the delivery of healthcare via information technology [1]. Ever since mobile phones provided internet access, the number of e‑Health projects has rapidly increased. Nevertheless, so far, it could be argued that systematic integration of e‑Health in cardiovascular disease management has yet to be established. Currently, remote monitoring of patients using mobile devices is not standard practice in most cardiology clinics.

This is in contrast with studies in which it is shown that e‑Health implementation will lead to a higher quality of care and may result in a reduction of costs [2]. Whereas the opinion and expectations of citizens, patients, healthcare insurers and especially administrators about e‑Health have been documented in the popular literature, the attitudes of physicians towards e‑Health have been less well evaluated. Literature describing the attitude of doctors towards e‑Health is scarcely available [3]. Therefore, the primary purpose of this study was to assess current application of e‑Health in routine clinical care and to identify both the expectations and perceived barriers to implementation of cardiovascular e‑Health applications.

## Methods

A tailored digital survey consisting of 30 questions was issued in November 2017. The complete survey is given in Appendix A of the online Electronic Supplementary Material. Respondents were cardiologists who were members of the Netherlands Society of Cardiology and for whom an e‑mail address was registered. The survey could be filled out digitally and was available for three consecutive weeks.

The first questions regarded the working environment and were divided into five categories: academic hospital, general hospital with a cardiothoracic surgery department (CTSD), general hospital without a CTSD, private clinic or ‘miscellaneous’. Furthermore respondents’ age category was registered as follows: 30–39 years, 40–49 years, 50–59 years or 60+ years.

Subsequently, questions were asked regarding five topics:

1. Personal use of smartphones and applications to track health data.
2. Digital, patient-driven communication between doctor and patients. E. g. through e‑mail, smartphone applications and/or a dedicated functionality within the electronic medical record (EMR).
3. Current use of e‑Health in clinical practice, such as e‑visits and remote monitoring.
4. Expectations surrounding e‑Health, subdivided into six statements about possible benefits or drawbacks of e‑Health, to which subjects could agree, partially agree (in the results taken together as ‘agree’), partially disagree and fully disagree (in the results section taken together as ‘disagree’).
5. Perceived barriers to implementation of e‑Health, subdivided into eight barriers to e‑Health implementation, about which respondents could indicate the level (1, almost not inhibiting to 5, very much inhibiting) to which this barrier was present in their clinical practice.

### Statistical analysis

Statistical analysis was performed in SPSS (IBM Corp. Released 2013. IBM SPSS Statistics for Windows, Version 22.0. Armonk, NY: IBM Corp.). Scores were given as median ± interquartile range. To evaluate correlations between baseline characteristics and e‑Health, scores were developed. One score (the expectation score) indicates the level to which a cardiologist expects e‑Health to succeed (high score indicates high expectations). The calculation of the score is given in Appendix B of the online Electronic Supplementary Material. The other score (barrier score) is equal to the sum of answered numbers in that section of the questionnaire. Linear regression was performed to analyse the association between baseline characteristics (hospital working, age category, using the smartphone to track health data and using e‑mail or smartphones to communicate with patients) and the expectation and the barrier score. A *p* value ≤0.05 was considered statistically significant. Because of the low number of respondents in the miscellaneous group (*n* = 3) and private practice group (*n* = 8), these respondents were excluded from further analysis.

## Results

In total, 255 cardiologists filled out the questionnaire (response rate 25%). Characteristics are given in Tab. 1. Of the respondents, 248 (97.3%) used communication apps and 94 (36.9%) respondents indicated they used their smartphone to track health data. Seventy-five (29.4%) respondents used e‑mail to communicate directly with patients and 146 (57.3%) answered that this should be possible in the near future. Lastly, 42 (16.5%) denoted the use of communication applications such as Siilo® (Siilo, Amsterdam, the Netherlands), Whatsapp® (Facebook, Mountain View, California, USA), Skype® (Microsoft, Palo Alto, California, USA) or Facebook® (Menlo Park, California, USA) for direct communication with patients or other healthcare providers.

**Table 1:** Number of respondents per hospital working and per age category

	*N* (%)
*working environment*	
academic hospital	51 (20.0)
general hospital, with CTSD	39 (15.3)
general hospital, no CTSD	154 (60.4)
private clinic	8 (3.1)
miscellaneous	3 (1.2)
*age category*	
30–39	55 (21.6)
40–49	101 (39.6)
50–59	76 (29.8)
≥60	23 (9.0)

*CTSD* cardiothoracic surgery department

### Current implementation in clinical practice

In total, 89.2% respondents indicated that some form of remote monitoring was being used in clinical practice. This was predominantly remote monitoring of ICDs or pacemakers (61.6%). E‑visits (17.2%), remote monitoring of vital signs (14.3%) and remote monitoring of loop recorders (12.3%) were less common in clinical practice. A total of 41% indicated that a patient portal was integrated into the EMR of their hospital and 20.0% indicated that it was possible to communicate with patients through the EMR. Furthermore, 16.0% indicated that a digital questionnaire was integrated and 9.4% said that a videoconferencing program was integrated in the EMR.

### Expectations on e‑Health

**Fig. 1:**
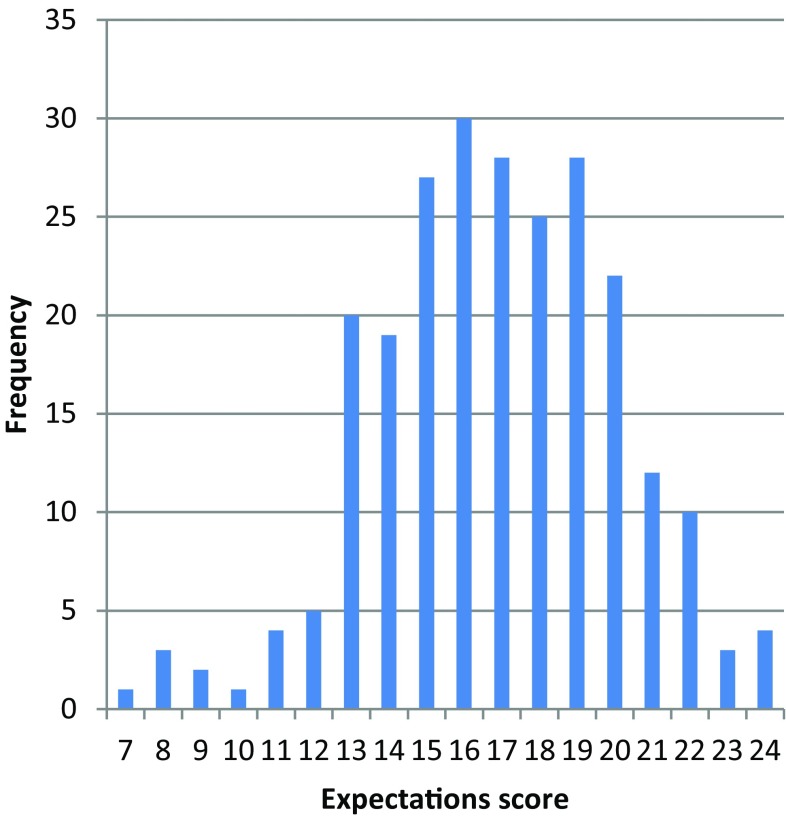
Expectation scores derived from questionnaire

A total of 228 (89.4%) of respondents indicated that e‑Health gives clinical benefit. Similarly, 230 (90.2%) thought that e‑Health will improve patient satisfaction and 214 (83.9%) expected e‑Health to help patients to be better informed about their condition and treatment options. In addition, 193 (75.7%) indicated that e‑Health may increase the workload. Of interest 155 (60.8%) indicated that e‑Health will reduce healthcare expenditure (despite the increase in workload). Lastly, 135 (52.9%) indicated that e‑Health could potentially impair the privacy of both patients and physicians. A histogram demonstrating the distribution of expectation scores is given in Fig. 1.

### Barriers to e‑Health

An overview of all eight barriers is given in Tab. 2. For three barriers, the majority of respondents indicated that they were inhibiting e‑Health implementation in their practice. Reimbursement issues (*n* = 170, 66.7%), lack of reliable devices by (*n* = 135, 52.9%) and lack of integration into EMRs (*n* = 177, 69.4%) were given as potential barriers. In contrast, 18 (7.1%) respondents indicated the unwillingness of patients and 48 (18.8%) denoted the potential inability of patients to work with smartphones as potential barriers to e‑Health implementation. Lastly, lack of scientific evidence, risk of data leaks and resistance of doctors and nurses were identified as barriers by 109 (42.7%), 90 (35.3%) and 105 (41.2%) respectively.

**Table 2:** Number of respondents and percentages per barrier

	yes	no	undecided
lack of scientific evidence	109 (42.7%)	72 (28.2%)	74 (29.0%)
lack of reimbursement	170 (66.7%)	30 (11.8%)	55 (21.6%)
lack of reliable devices	135 (52.9%)	51 (20.0%)	69 (27.1%)
patients are unwilling	18 (7.1%)	147 (57.6%)	90 (35.3%)
lack of data integration in EMRs	177 (69.4%)	37 (14.5%)	41 (16.1%)
patients are unable to use smartphones	48 (18.8%)	126 (49.4%)	81 (31.8%)
risk of data breach	90 (35.3%)	88 (34.5%)	77 (30.2%)
resistance of doctors and nurses	105 (41.2%)	66 (25.9%)	84 (32.9%)

*EMR* electronic medical records

### Correlation between baseline characteristics and expectations and perceived barriers

**Fig. 2:**
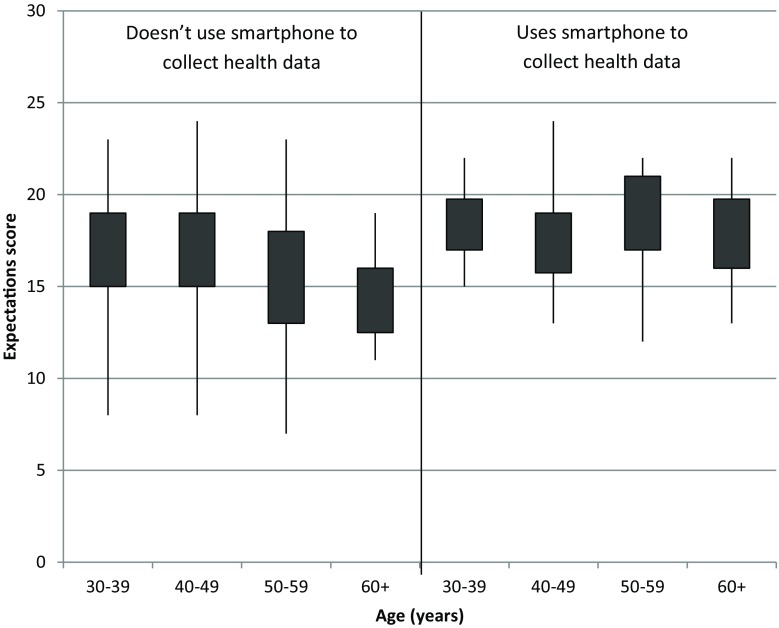
Expectation score per age and tracking health data

**Fig. 3:**
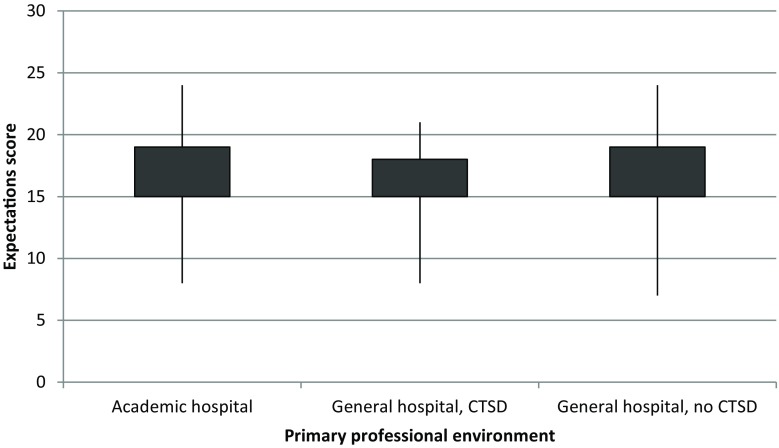
Expectation score per type of hospital (*CTSD* cardiothoracic surgery department)

A linear regression model showed that age category (−0.224; *p* < 0.001) tracking health data on the smartphone (0.273; *p* < 0.001), e‑mailing with patients (0.144; *p* = 0.029) and using communication apps to communicate with patients (0.205; *p* = 0.001) were all associated with the expectation score. Box plots of scores are shown in Figs. 2 and 3. However, none of these variables were associated with the barrier score.

## Discussion

In this study, implementation of e‑Health in cardiology practice was assessed, as well as expectations of e‑Health and barriers for implementation in clinical practice as perceived by cardiologists. The main findings were that 89% of respondents indicated they saw e‑Health as clinically beneficial, that the age of the respondent was a negative predictor and personal use of smartphones was a positive predictor of having high expectations and that lack of data integration in EMRs was identified as barrier by 69.4% of respondents.

### Implementation in current practice

In this study, 89.2% of respondents indicated that their patients were followed using some form of remote monitoring, which was predominantly remote monitoring of active devices such as pacemakers or ICDs. E‑visits, remote monitoring of vital signs and loop recorders were less common. This is most likely due to a combination of level of scientific evidence and history. Remote monitoring of pacemakers was already introduced in the early 1970s and has been common since the 1990s [4, 5]. It has been demonstrated that follow-up with remote monitoring of ICDs can help to reduce all-cause mortality and reduces the risk of inappropriate shocks [6]. On the contrary, remote monitoring of vital signs via smartphones was introduced in the late 2000s and its clinical benefit is, dependent on the specific patient population, less well established in the scientific literature. The first results of pilot projects show promising findings and therefore more research in trials with larger sample sizes to investigate the effect of remote monitoring of vital signs on clinical outcomes is justified.

In this study, 16.5% of respondents indicated they used apps to communicate with patients or other healthcare providers. This percentage may be low due to the privacy issues related to commonly used communication apps such as Facebook Messenger and Whatsapp. In fact, the Dutch Data Protection Authority recommend not to use these apps in doctor-doctor or doctor-patient communication [7].

### Expectations

More than 80% of respondents indicated that e‑Health may be clinically beneficial, improve patient satisfaction and will help to improve patient information. In the current study, age was a negative predictor of e‑Health optimism and personal use of smartphones was a positive predictor. As mentioned, evidence about the expectations of physicians is scarcely available. One recent study (2017) in 930 physicians in the Barcelona region, Spain, found that physicians were generally in favour of e‑Health implementation, giving it a 7.4 on a 1–10 scale [3]. The authors also report that age and personal use of smartphones were important predictors of optimism about e‑Health. Interestingly, although the study population was different, they found similar percentages for using e‑mail, smartphone apps or social media to communicate with patients [3]. In the current study, age was negatively associated with e‑Health expectations. However, this effect was not observed in the group who used their smartphone to track health data. This study did not assess the reason for this phenomenon, but it could be that respondents who use their smartphone to track health data are generally more optimistic towards the possibilities of smartphone technology, regardless of age. This reflects their expectations towards e‑Health. Lastly, a correlation between expectations and the type of hospital where a respondent was working was not present.

### Barriers

Three subjects were perceived as major barriers by respondents: reimbursement issues, lack of reliable devices and insufficient data integration in EMRs. For the first item, it is important to note that this questionnaire was issued in 2017. As of 1 January 2018, e‑visits and telemonitoring are, conditionally, reimbursed in the Netherlands [8]. Therefore, it is questionable whether lack of reimbursement would still be identified as a barrier by 52%, as some forms of e‑Health are now reimbursed. Lack of reliable devices was recognised in the literature as an important barrier [9–11]. In a recent study 107 apps for hypertension management were investigated. It was found that 14% of these applications claimed to be able to measure blood pressure by using the smartphone only. Some of these applications had over a million downloads. However, none of these devices were validated against a gold standard [9]. In another study, it was demonstrated that a diabetes application was likely to give a recommendation for an inappropriate insulin dosage to its user [12]. For the safety of patients, it is important that app stores are regularly screened for applications that turn the smartphone into a medical device. It could be argued that these applications should be validated against a gold standard and results should be published in a peer-reviewed journal before this application is available for download.

In this study, most respondents indicated that e‑Health applications are not integrated into EMRs. Furthermore, it was found that the majority of physicians identified this lack of data integration into EMRs as a barrier to successful e‑Health implementation. Currently, there are over 45,000 m-Health apps in the app store [13]. It will be practically impossible for physicians to log into these accounts while seeing a patient at the outpatient clinic. Therefore, data integration should be a key priority for e‑Health innovations in cardiology.

In total, 75% of respondents indicated that e‑Health will increase workload. This study did not investigate the reasons behind respondents’ answers. Evidence on the workload of e‑Health is limited. There is evidence that remote monitoring of ICDs decreased office visits and rehospitalisation [14]. However, evidence in other patient populations and other forms of remote monitoring is inconclusive. One possible explanation could be the lack of data integration into different EHRs. With multiple systems being used, the input of data into multiple systems is time-consuming and therefore not cost-effective. Better data integration could improve this [15]. Whether or not e‑Health decreases workload should therefore be carefully monitored in future initiatives.

### Future

The results of this study indicate that the majority of the responding cardiologists have a positive feeling about the future of e‑Health. However, some serious barriers have to be overcome before e‑Health will be common in daily practice. Furthermore, it is important to recognise that e‑Health is not a simple translation from routine care by adding some electronic devices. The widespread implementation of e‑Health in combination with patient owned EMRs following the patient during his or life will really change the way we provide care [15]. It can be expected that this transition will also implicate that healthcare will move in the direction of taking care of health [15].

### Limitations

Two limitations have to be taken into account when interpreting these data. First, this questionnaire had a limited response rate of 25%. Results might therefore be at risk of selection bias. Second, the scoring system for the questionnaire has not been previously validated. Therefore, there is some level of uncertainty as to whether the scores actually reflect cardiologists’ general positivism against e‑Health. It is emphasised that these scores should be seen as a level of agreement with the statements made in the questionnaire.

## Conclusion

Cardiologists are in general optimistic about the possibilities of e‑Health, but identify lack of data integration into the EMR, reimbursement issues and lack of reliable devices as major barriers. Age and personal use of smartphones are predictors of expectations of e‑Health, but the working environment is not.

## Caption Electronic Supplementary Material

Appendix A A is an English translation of the Dutch questionnaire that was given to cardiologists.

Appendix B provides insight in how the expectations score, given in the results section, was calculated out of the results of the questionnaire
