# Supplementary material for: Expectations and perceived barriers to widespread implementation of e‑Health in cardiology practice: Results from a national survey in the Netherlands
Source: Neth Heart J. 2018 Nov 28;27(1):18–23. doi: 10.1007/s12471-018-1199-9 (PMC6311154; doi:10.1007/s12471-018-1199-9)
Supplement: Supplementary file 1 — Appendix A A is an English translation of the Dutch questionnaire that was given to cardiologists. [file 12471_2018_1199_MOESM1_ESM.docx]

**Questionnaire, translated from Dutch**

1. In which healthcare enterprise do you work?
   - Private practice
   - General hospital, with cardiothoracic surgery department
   - General hospital, without cardiothoracic surgery department
   - Academic medical center
   - Other, namely:
   - I prefer not to answer this question
2. What is your age (in between 30-39, 40-49, 50-59, 60+)?
   - 30-39
   - 40-49
   - 50-59
   - 60+
   - I prefer not to answer this question
3. Do you own a smartphone?
   - Yes
   - No
   - I prefer not to answer this question
4. Do you use communication apps (Whatsapp, Facebook, Twitter, etc.) on your smartphone?
   - Yes
   - No
   - I prefer not to answer this question
5. Do you use your smartphone to track health data (blood pressure, step counter, ECG device)?
   - Yes
   - No
   - I prefer not to answer this question

**Communication between your patient and you**

1. Can patients send an e-mail to you directly, without communication via a secretary?
   - Yes
   - No
2. If it fits the therapeutic relationship, do you believe that direct e-mail contact should be possible?
   - Yes
   - No
3. Do you use communication apps like Whatsapp, Siilo, Skype of Facebook to communicate with your patient?
   - Yes
   - No
4. What percentage of your patients (do you estimate) would want to use e-visits (outpatient clinic visits in which the patient stays at home and calls the doctor via a videoconferencing system)?
   - 0-19
   - 20-39
   - 40-59
   - 60-79
   - 80-100

**In your practice**

1. Is it possible, in your practice, to make an appointment via a web portal?
   - Yes
   - No
2. Do you use e-visits in your practice?
   - Yes
   - No
3. Are there patients in your practice who are using a remote monitoring system (e.g. patients who are using remote monitoring of their ICD)?
   - Yes, remote monitoring of ICDs
   - Yes, remote monitoring of loop recorders
   - Yes, symptom registration (e.g. dyspnoea, orthopnoea) via internet
   - Yes, remote monitoring of vital signs (blood pressure, weight) for heart failure patients
   - Yes, other, namely:
   - No
4. Is the electronic medical record in your practice equipped with a patient portal?

- Yes, patients can only read information via the patient portal
- Yes, patients can look up information, but can also make/change appointments
- Yes, patients can look up information, but can also make/change appointments and send messages to their treating physician
- An EMR is not used in my practice
- No

1. Is one of the following applications integrated in the electronic medical record that is used in your practice?

- E-Visit
- Sending messages to the patient
- Receive messages from the patient
- Patient portal
- Digital questionnaire, preceding an outpatient clinic visit
- Results of telemonitoring are integrated in the EMR
- There are no applications integrated in the EMR
- An EMR is not used in my practice

**The following statements are about the usability of e-Health. Could you please indicate if you totally agree, partially agree, partially disagree or totally disagree with the following statements?**

|  | Totally agree | Partially agree | Partially disagree | Totally disagree |
| --- | --- | --- | --- | --- |
| e-Health is clinically beneficial |  |  |  |  |
| e-Health will cut healthcare costs |  |  |  |  |
| e-Health will enhance patient satisfaction |  |  |  |  |
| By using e-Health patients will be better informed |  |  |  |  |
| e-Health threatens both doctors’ and patients’ privacy |  |  |  |  |
| e-Health will increase workload for doctors and nurses |  |  |  |  |
| e-Health will contribute to a situation in which a hospital is 24/7 available for non-emergency care |  |  |  |  |
| It is a good thing that a hospital will be 24/7 available for non-emergency care |  |  |  |  |

**E-health implementation**

Factors that determine e-Health implementation in your practice (1-absolutely not; 5- very much):

|  | 1 | 2 | 3 | 4 | 5 |
| --- | --- | --- | --- | --- | --- |
| The lack of scientific evidence |  |  |  |  |  |
| The lack of reimbursement |  |  |  |  |  |
| Lack of reliable devices |  |  |  |  |  |
| Patients do not want to use e-Health |  |  |  |  |  |
| The lack of data integration of commercial devices into the electronic medical record |  |  |  |  |  |
| Patients are not capable of using smartphones |  |  |  |  |  |
| Risk of a data leakage is to high |  |  |  |  |  |
| Doctors and nurses do not want it |  |  |  |  |  |
