# Supplementary material for: Expectations and perceived barriers to widespread implementation of e‑Health in cardiology practice: Results from a national survey in the Netherlands
Source: Neth Heart J. 2018 Nov 28;27(1):18–23. doi: 10.1007/s12471-018-1199-9 (PMC6311154; doi:10.1007/s12471-018-1199-9)
Supplement: Supplementary file 2 — Appendix B provides insight in how the expectations score, given in the results section, was calculated out of the results of the questionnaire [file 12471_2018_1199_MOESM2_ESM.docx]

Appendix B, calculation of the expectations score

|  | Totally agree | Partially agree | Partially disagree | Totally disagree |
| --- | --- | --- | --- | --- |
| e-Health is clinically beneficial | 1 | 1 | 0 | 0 |
| e-Health will cut healthcare costs | 1 | 1 | 0 | 0 |
| e-Health will enhance patient satisfaction | 1 | 1 | 0 | 0 |
| By using e-Health patients will be better informed | 1 | 1 | 0 | 0 |
| e-Health threatens both doctors’ and patients’ privacy | 0 | 0 | 1 | 1 |
| e-Health will lead to a higher workload | 0 | 0 | 1 | 1 |
| e-Health will contribute to a situation in which a hospital is 24/7 available for non-emergency care | 1 | 1 | 0 | 0 |
| It is a good thing that a hospital will be 24/7 available for non-emergency care | 1 | 1 | 0 | 0 |

The “expectations score” was the sum of all answers given.
